# Low-level laser therapy associated to a resistance training protocol on bone tissue in diabetic rats

**DOI:** 10.1590/2359-3997000000190

**Published:** 2016-08-23

**Authors:** Tatiane Lopes Patrocínio-Silva, André Moreira Fogaça de Souza, Raul Loppi Goulart, Carolina Fuirini Pegorari, Jussan Rodrigues Oliveira, Kelly Rossetti Fernandes, Angela Maria Paiva Magri, Rosa Maria Rodrigues Pereira, Daniel Araki Ribeiro, Márcia Regina Nagaoka, Ana Claudia Muniz Rennó

**Affiliations:** 1 Departamento de Biotecnologia Universidade Federal de São Carlos São Carlos SP Brasil Departamento de Biotecnologia, Universidade Federal de São Carlos (UFSCar), São Carlos, SP, Brasil; 2 Departamento de Biociências Universidade Federal de São Paulo Santos SP Brasil Departamento de Biociências, Universidade Federal de São Paulo (Unifesp), Santos, SP, Brasil; 3 Departamento de Medicina Universidade de São Paulo São Paulo SP Brasil Departamento de Medicina, Universidade de São Paulo (USP), São Paulo, SP, Brasil

**Keywords:** Low-level laser therapy, resistance training, diabetes mellitus, bone

## Abstract

**Objective:**

The present study aimed to evaluate the in vivo response of a resistance training and low-level laser therapy (LLLT) on tibias and femurs of rats with diabetes mellitus (DM).

**Materials and methods:**

Forty male Wistar rats were randomly distributed into four experimental groups: control group (CG), diabetic group (DG), diabetic trained group (TG) and diabetic trained and laser irradiated group (TLG). DM was induced by streptozotocin (STZ) and after two weeks laser and resistance training started, performed for 24 sessions, during eight weeks. At the end of the experiment, animals were euthanized and tibias and femurs were removed for analysis. Histological, histomorphometrical, immunohistochemistry and mechanical analyses were performed.

**Results:**

Trained groups, with or without laser irradiation, showed increased cortical area, bone density and biomechanical properties. The immunohistochemical analysis revealed that TG and TLG demonstrated an increased RUNX2 expression. RANK-L immunoexpression was similar for all experimental groups.

**Conclusion:**

In conclusion, it can be suggested that the resistance exercise program stimulated bone metabolism, culminating in increased cortical tibial area, bone mineral content, bone mineral density and biomechanical properties. Furthermore, the association of physical exercises and LLLT produced higher values for bone mineral content and stiffness. Consequently, these data highlight the potential of physical exercise in the management of bone loss due to DM and the possible extra osteogenic stimulus offered by lasertherapy. Further long-term studies should be carried out to provide additional information.

## INTRODUCTION

Diabetes mellitus (DM) is a chronic disease characterized by hyperglycemia resulting from deficits of insulin secretion, insulin action, or both (1). It affects 6 to 8% of the population worldwide and it leads to a variety of complications that includes nephropathy, retinopathy and cardiovascular disease (2).

In addition, many authors reported changes in bone metabolism, with a decreased osteoblast activity and increased osteoclast proliferation, resulting in lower bone mineral density (BMD) (3). Miazgowski and Czekalski (4) demonstrated that diabetic patients had significantly lower BMD and an increased incidence rate of osteopenia and osteoporosis. Consequently, due to the lower BMD bone fractures are found to have an increased incidence in the presence of both types of diabetes (5). Moreover, lower biomechanical resistance, deficiency in bone mineralization after a fracture and decreased bone calcium and phosphate content were observed in induced diabetic animals (6).

Since DM has become one of the most important health problems, it is important to develop effective treatments able to stimulate bone formation. One of the most promising treatments to manage bone loss in the clinical situation of DM is physical exercise programs (7).

Physical exercises acts trough a mechanical loading via weight bearing and induces temporary bone deformation, which provide an adequate stimulus to activate bone formation and increase BMD (8). Some studies have showed that physical exercises can manage bone mass loss in DM patients (9). Gomes and cols. (10) observed that physical training increased the serum IGF-1 level in the diabetic trained rats and increased tibial length, total area and bone mineral content in these animals compared to control.

Similarly, some attention has been given to the effect of low-level laser irradiation (LLLT) on biological tissues, including bone. Studies have showed that LLLT is able to modulate cell metabolism and stimulate mitochondrial respiration, particularly in the production of molecular oxygen and ATP synthesis (11-13). These effects produce an increase in DNA, RNA and cell cycle regulatory protein synthesis, which stimulate cell proliferation (11,14).

In bone, LLLT has a stimulatory effect and can increase cell proliferation and accelerating fracture consolidation (15). Moreover, LLLT has stimulatory effects on bone mass in osteoporotic and diabetic rats (12,15). Bayat and cols. (12) observed that a He-Ne laser (632.8 nm, 10 mW) produced a significantly improvement in the tibias biomechanical properties and greater bone lamella meshwork in diabetic rats.

Although the encouraging data on the use of physical exercises and LLLT in bone tissue, the effects of both therapies, used in association, in diabetic rats have not been investigated yet. Before both therapies can be used with confidence as a therapeutic modality in bone tissue in the presence of DM, it is necessary to investigate the effects and dose-response characteristics of these treatments *in vivo* in studies to determine its safety and efficacy. In view of the aforementioned, it was hypothesized that laser irradiation could optimize the stimulatory effect of exercises in bone tissue in diabetic rats, increasing bone mass and bone biomechanical properties. Consequently, the present study aimed to evaluate the *in vivo* response of a resistance training and LLLT on tibias and femurs of induced DM rats. To this end, Wistar rats were distributed into four different groups named (control group, diabetic group, diabetic trained group and diabetic trained and laser irradiated group) and the bone response was evaluated by means of a histological analysis, immunohistochemistry, densitometry and biomechanical analysis.

## MATERIALS AND METHODS

### Experimental design

Forty male Wistar rats (aged 8 weeks and weighing 290 ± 6.8 g) were used in this study. They were maintained under controlled temperature (22 ± 2^o^C), light-dark periods of 12 hours and with free access to water and commercial diet. All animal handling and procedures were strictly conducted according to the Guiding Principles for the Care and Use of Laboratory Animals. This study was approved by the Ethical Committee of the Federal University of São Paulo (2010/145).

Rats were randomly distributed into four groups (n = 10 each group): control group (CG), diabetic group (DG), diabetic trained group (TG) and diabetic trained and laser irradiated group (TLG).

### Induction of DM and body mass evaluation

DM was induced using a single dose of intravenous injection of pancreatic β-cell toxin STZ (Sigma-Aldrich Corp.^®^, St. Louis, MO, USA) (60 mg/kg of body weight) dissolved in sterile citrate buffer into the penile vein (16).

Rats of CG received an injection of citrate buffer. Two weeks after the procedure, glucose level was measure and DM was defined as blood glucose concentration greater than 300 mg/dL in a blood sample (Accutrend^®^ Plus, Roche-Diagnostics GmbH, Mannheim, Germany) (17). To determine the level of blood glucose, animals were immobilized manually and a small cut was made on the tail to obtained 25 ul of blood. These procedures were repeated once a week throughout the study. Furthermore, the body mass was monitored weekly by a weighing-machine (Kern^®^ 440-21A; Balingen-Frommern German). In addition, initial body mass (IBM) (considered as the body mass 14 days after the induction of DM) and final body mass (FBM) were considered as to determine the maximal load supported by each animal during the physical test in the beginning and in the end of the experiment.

### Resistance training protocol and determination of the maximal load

The resistance protocol was consisted of a climbing exercise using a training support ladder apparatus (1.1 x 0.18 m, 2 cm grid, 80° inclination) with a housing chamber (20 x 20 x 20 cm) at the top of the ladder. The size of the ladder induced the animals to perform eight to twelve dynamic movements per climb. The load apparatus was secured to the tail by wrapping the proximal portion of the tail with a self-adhesive foam strip. A Velcro strap was wrapped around the foam strip and fastened. If necessary, a stimulus with tweezers was applied to the animal’s tail to initiate movement (3). The training started with 2 familiarization sessions with 24 hours between them, without any weight attached to the tail of the animals. These sections had the aim of teaching the rat to climb the ladder and were consisted of 3 consecutive climbings to the top, with an interval of 60 seconds of resting.

One day after this familiarization, the test to determine the maximal load supported by each animal was performed. This consisted of four to eight ladder climbs while carrying progressively heavier loads. For the initial climb, the load carried was 75% of the animal’s body mass. After this, an additional 30 g weight was added, until a load was reached with which the rat could not climb the entire length of the ladder. Failure was determined when the animal could not progress up the ladder after three successive stimuli to the tail. The highest load successfully carried the entire length of the ladder was considered the rat’s maximal carrying capacity for that training session. The next training session consisted of four ladder climbs with 50%, 75%, 90%, and 100% of the rat’s previous maximal carrying capacity, determined in the previous session. During subsequent ladder climbs, an additional 30 g load was added until a new maximal carrying capacity was determined. The test to determine the maximal load was repeated after the 8 week-resistance training protocol. The resistance training protocol was performed three times per week for eight weeks. The animals performed 8 – 10 movements during each session of training.

### Low-level laser irradiation

Laser treatment was performed 3 times per week, for 24 sessions, with an interval of 24 h between sessions. A low-energy Ga-Al-As laser (Photon lase III, DMC Equipment, São Carlos, SP, Brazil), 808 nm, 100 mW, continuous wavelength, 0.028 cm^2^, 3.57 W/cm^2^, 33 s, 120 J/cm^2^, 3.3 J were used. The irradiation was performed after the resistance exercise protocol, at one point in the middle region of both tibias and femurs, through the punctual contact technique. Twenty four hours after the last treatment session, rats were euthanized by CO_2_ inhalation and bones were removed for analysis.

### Histological procedures

Histology was performed in the left tibias of animals. After the harvesting, specimens were fixated in 10% formaline for 1 day, decalcified in 4% EDTA (Merck) and embedded in paraffin blocks. Thin sections (5 µm) were prepared perpendicular to the medial-lateral drilling axis of samples using a microtome (Leica Microsystems SP 1600, Nussloch, Germany). At least, three sections of each specimen were stained with hematoxylin and eosin (H.E stain, Merck) (15).

### Histomorphometrical evaluation

Histomorphometry of the total area (TTA) and cortical area (CTA) were performed using a light microscope (Leica Microsystems AG, Wetzlar, Germany), at a magnification of 2.5 x, equipped with a digital camera (AxioCam HRc, Carl Zeiss, Germany) and a computer-based image analysis techniques (Leica^®^ Qwin Pro-image analysis system, Wetzlar, Germany) (18). Two experienced observers (TLPS and DAR) performed the evaluation in a blinded manner.

### Immunohistochemistry

Paraffin was removed with xylene from serial sections of 4 μm and the sections were rehydrated in graded ethanol, then pretreated in a microwave with 0.01 M citric acid buffer (pH 6) for three cycles of 5 min each at 850W for antigen retrieval. The material was pre-incubated with 0.3% hydrogen peroxide in phosphate-buffered saline (PBS) solution for 5 min for inactivation of endogenous peroxidase and then blocked with 5% normal goat serum in PBS solution for 10 min. The specimens were then incubated with anti-RUNX2 and anti-RANK monoclonal primary antibody (Santa Cruz Biotechnology, USA) at a concentration of 1:100. Incubation was carried out overnight at 4°C within the refrigerator. The sections were then incubated with biotin conjugated secondary antibody anti-rabbit IgG (Vector Laboratories, Burlingame, CA, USA) at a concentration of 1:200 in PBS for 1 h. The sections were washed twice with PBS followed by the application of performed avidin biotin complex conjugated to peroxidase (Vector Laboratories) for 45 min. The bound complexes were visualized by the application of a 0.05% solution of 3-3’-diaminobenzidine solution and counterstained with Harris hematoxylin. For control studies of the antibodies, the serial sections were treated with rabbit IgG (Vector Laboratories) at a concentration of 1:200 in place of the primary antibody. Additionally, internal positive controls were performed with each staining bath (19).

### Mechanical test

Biomechanical properties of the right tibias were determined by a three-point bending test in an Instron^®^ Universal Testing Machine (USA, 4444 model, 1 kN load cell). Tibias were placed on a 3.8 cm-long metal device, which provides a 1.8-cm-distant double support on the bone diaphysis. The load cell was perpendicularly positioned at the middle point of the bone. A 5 N pre-load was applied in order to avoid specimen sliding. Finally, the bending force was applied at a constant deformation rate of 0.5 cm/min until fracture occurred. From the load-deformation curve, the maximum strength (kN), toughness (J), resilience (J), stiffness (N/mm) and fracture force (kN) were obtained (12).

### Densitometry

To measure bone mineral content (BMC) and bone mineral density (BMD) of the right femur, densitometry analysis was carried out by using a densitometer (DEXA Hologic Inc Discovery model-Belford, MA, USA) and specific software for small animals (20).

### Statistical analysis

All variables were organized into mean and standard deviation. The distribution of all variables was tested for normality by using Shapiro–Wilk’s W test. For the variable manifesting a normal distribution, comparisons among the groups were made via one-way analysis of variance (ANOVA), complemented by Tukey post-hoc analysis. STATISTICA version 7.0 (data analysis software system – StatSoft Inc.) was used to carry out statistics analysis. Values of *p* ≤ 0.05 were considered statistically significant.

## RESULTS

### General observation of the experimental animals

From the 48 animals available for this study, two animals were lost due to a respiratory depression induced by the anesthesia before the procedure to induce DM. In addition, six animals were lost after the surgical procedure of DM induction. The other animals rapidly returned to their normal diet and no post-operative complications were observed.

### Sample characterization

The FBM was higher in the CG (331.1 ± 28.9 g) compared to the diabetic animals (p < 0.0001 for DG (191.4 ± 8.8 g), TG (246.0 ± 29.7 g) and TLG (255.2 ± 32.6 g)). No other difference was found in the IBM (Table 1). Furthermore, TG (246.0 ± 29.7 g) and TLG (255.2 ± 32.6 g) demonstrated a higher FBM when compared to DG (191.4 ± 8.8 g) (p = 0.0008 and p < 0.0001, respectively) (Table 1). The level of blood glucose was significantly lower in the CG (256.1 ± 49.3 mg/dL – IGL; 272.0 ± 14.3 mg/dL – FGL) compared to all the diabetic groups, both at the beginning (p = 0.0003, p < 0.0001 and p < 0.0007 for DG (393.2 ± 51.0 mg/dL), TG (415.5 ± 79.2 mg/dL) and TLG (379.2 ± 74.8 mg/dL), respectively) and the end of the experiment (p < 0.0001, < 0.0001 and 0.0005 for DG (509.3 ± 56.3 mg/dL), TG (396.4 ± 54.6 mg/dL) and TLG (377.4 ± 55.4 mg/dL), respectively). Furthermore, final glucose level (FGL) of the TG (396.4 ± 54.6 mg/dL) and TLG (377.4 ± 55.4 mg/dL) was significantly lower compared to DG (509.3 ± 56.3 mg/dL) (p = 0.0002 and p < 0.0001, respectively) (Table 1). No other difference was observed on glucose levels.

**Table 1:** Results of body mass and glucose levels

Variables	IBM (g)	FBM (g)	IGL (mg/dL)	FGL (mg/dL)
CG	305.0 ± 31.7	331.1 ± 28.9	256.1 ± 49.3	272.0 ± 14.3
DG	278.7 ± 32.8	191.4 ± 8.8^a^	393.2 ± 51.0^d^	509.3 ± 56.3^a^
TG	269.4 ± 22.4	246.0 ± 29.7^a,b^	415.5 ± 79.2^a^	396.4 ± 54.6^a,g^
TLG	267.9 ± 31.9	255.2 ± 32.6^a,c^	379.2 ± 74.8^e^	377.4 ± 55.4^c,f^

CG: control group; DG: diabetic group; TG: diabetic trained group; TLG: diabetic trained and laser irradiated group; IBM: initial body mass; FBM: final body mass; IGL: initial glucose level; FGL: final glucose level. ^a^ vs. CG, p < 0.0001; ^b^ vs. DG, p = 0.0008; ^c^ vs. DG, p < 0.0001; ^d^ vs. CG, p = 0.0003; ^e^ vs. CG, p = 0.0007; ^f^ vs. CG, p = 0.0005; ^g^ vs DG, p = 0.0002.

### Histomorphometrical evaluation

**Figure 1:**
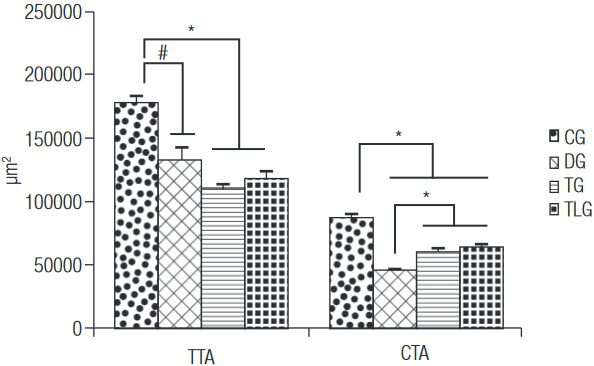
Mean and standard deviation of the histomorphometrical evaluation.

TTA: total tibial area; CTA: cortical tibial area; CG: control group; DG: diabetic group; TG: diabetic trained group; TLG: diabetic trained and laser irradiated group. ^#^ p = 0.0001, * p < 0.0001.

Figure 1 shows the data related to the morphometric analysis, TTA of the CG (178573 ± 4990.15 µm^2^) was higher compared to the other groups (p = 0.0001, p < 0.001 and p < 0.001 for DG (132274 ± 10412.75 µm^2^), TG (110519 ± 5164.64 µm^2^) and TLG (118428 ± 5706.23 µm^2^)). No other difference was found. Furthermore, CG (87883 ± 2483.12 µm^2^) showed higher values of CTA than DG (46104 ± 957.48 µm^2^), TG (60697 ± 2094.97 µm^2^) and TLG (64064 ± 1895.96 µm^2^) (p < 0.0001 for DG, TG and TLG). Moreover, trained groups, with or without laser irradiation, showed higher values for this variable compared to diabetic control animals (Figure 1).

### Immunohistochemistry

**Figure 2:**
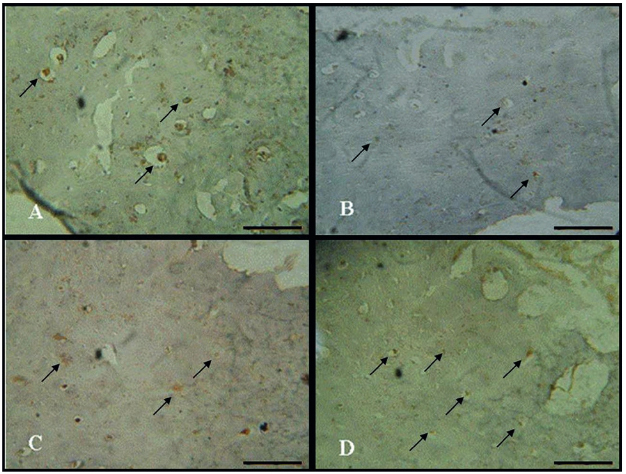
RUNX2 immunoexpression. (A) Control group; (B) Diabetic group; (C) Diabetic trained group and (D) Diabetic trained and laser irradiated group. Scale bar 100 µm.

RUNX2 immunoexpression was detected in the cytoplasm of bone cells. In the CG, a high expression was detected (Figure 2A). However, in the diabetic control animals, a decreased RUNX2 immunoexpression was noticed when compared to CG (Figure 2B). Trained groups, with and without laser irradiation presented higher RUNX2 immunoexpression compared to DG (Figure 2C and D).

**Figure 3:**
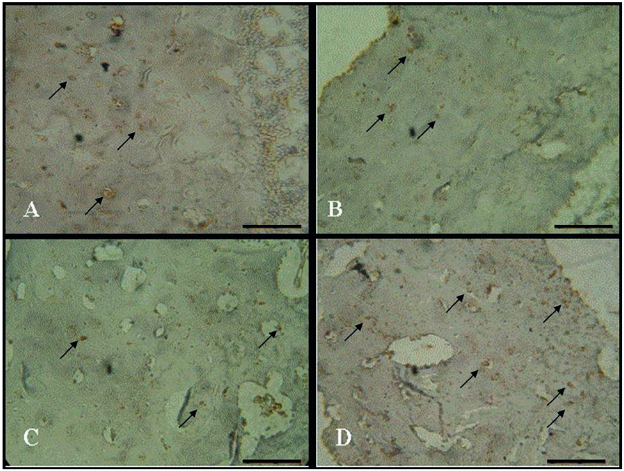
RANK-L immunoexpression in rat tibia. (A) Control group; (B) Diabetic group; (C) Diabetic trained group and (D) Diabetic trained and laser irradiated group. Scale bar 100 µm.

RANK-L immunoexpression was weakly detected in bone cells, without remarkable differences between groups (Figure 3).

### Biomechanical analysis

Table 2 shows the values of the biomechanical properties. Maximum strength was significantly lower in DG (0.055 ± 0.001 kN) and TLG (0.060 ± 0.004 kN) compared to CG (0.073 ± 0.004 kN) (p = 0.0035 and p = 0.0353, respectively). Mean values of toughness were significantly lower in all diabetic groups (DG (0.049 ± 0.008 J), TG (0.051 ± 0.008 J) and TLG (0.052 ± 0.003 J)) compared with the CG (0.079 ± 0.007 J) (p = 0.0174, p = 0.0277 and p = 0.0292, respectively). The CG (0.028 ± 0.004 J) revealed a significant increase compared to TLG (0.018 ± 0.001 J) (p = 0.0328) for resilience. Stiffness and fracture force were higher in CG (stiffness: 161.863 ± 1.798 N/mm; fracture force: 0.054 ± 0.001 kN)) compared to DG (stiffness: 138.675 ± 0.944 N/mm; fracture force: 0.030 ± 0.001 kN) (stiffness: p = 0.0217 and fracture force: p = 0.0011).

**Table 2:** Results of the biomechanichal analysis

	Maximum	Toughness	Resilience	Stiffness	Fracture
	**strength (kN)**	**(J)**	**(J)**	**(N/mm)**	**force (kN)**
CG	0.073 ± 0.004	0.079 ± 0.007	0.028 ± 0.004	161.863 ± 1.798	0.054 ± 0.001
DG	0.055 ± 0.001^a^	0.049 ± 0.008^c^	0.024 ± 0.002	138.675 ± 0.944^g^	0.030 ± 0.001^h^
TG	0.062 ± 0.003	0.051 ± 0.008^d^	0.022 ± 0.002	142.944 ± 7.268	0.043 ± 0.004
TLG	0.060 ± 0.004^b^	0.052 ± 0.003^e^	0.018 ± 0.001^f^	154.607 ± 5.801	0.042 ± 0.005

CG: control group; DG: diabetic group; TG: diabetic trained group; TLG: diabetic trained and laser irradiated group. ^a^ vs. CG, p = 0.0035; ^b^ vs. CG, p = 0.0353; ^c^ vs. CG, p = 0.0174; ^d^ vs. CG, p = 0.0277; ^e^ vs. CG, p = 0.0292; ^f^ vs. CG, p = 0.0328; ^g^ vs. CG, p = 0.0217; ^h^ vs. CG, p = 0.0011.

### Bone densitometry

**Figure 4:**
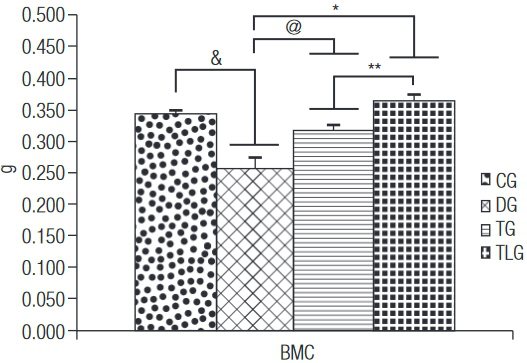
Bone densitometry.

BMC: bone mineral content; CG: control group; DG: diabetic group; TG: diabetic trained group; TLG: diabetic trained and laser irradiated group. ^&^ p = 0.0005, ^@^ p = 0.0204, * p < 0.0001, ** p = 0.0263.

**Figure 5:**
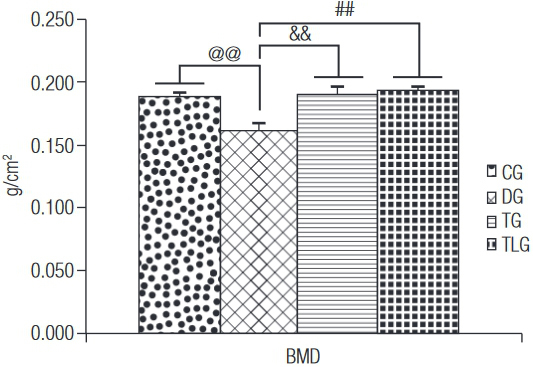
Bone densitometry.

BMD: bone mineral density; CG: control group; DG: diabetic group; TG: diabetic trained group; TLG: diabetic trained and laser irradiated group. ^@@^ p = 0.0061, ^&&^ p = 0.0023, ^##^ p = 0.0006.

The densitometric analysis demonstrated that CG and diabetic trained groups (with or without laser) showed a higher BMC compared to DG (p = 0.0005, 0.0204, < 0.0001 for CG, TG and TLG) (Figure 4). Moreover, BMC of TLG was higher compared to TG (p = 0.0263) (Figure 4). Figure 5 shows that BMD of DG was significantly lower compared to the other groups (p = 0.0061, 0.0023 and 0.0006 for CG, TG and TGL, respectively).

## DISCUSSION

This study aimed to evaluate the *in vivo* bone tissue response of a resistance training, associated or not with LLLT in diabetic rats. The main findings showed that both trained groups demonstrated a higher cortical area, higher RUNX2 immunoexpression and increased fracture force, BMC and BMD compared to diabetic control. Furthermore, it seems that LLLT was able of increasing stiffness and BMD in the trained animals.

A considerable body of evidences demonstrated dysfunctions in bone tissue related to DM, with the decrease in bone density and increase in fracture incidence (8). In recent years, the identification of effective treatment strategies is the key to minimizing all the alterations related to DM, including resistance physical exercises (8,21). Bone tissue responds to external forces and it is able to adapt to endure loading conditions (22). Over time, this process leads to an increase in bone density and biomechanical properties in normal and pathological bone conditions, such as osteoporosis and DM (15,23). Moreover, many authors evidenced the osteogenic potential of LLLT (12,13,15). It is well established that, laser light is able of interacting with bone tissue, modulating bone cell biochemical reactions and stimulating mitochondrial respiration. The modifications in cell metabolism lead a higher production of molecular oxygen and ATP synthesis. Furthermore, evidences suggest that LLLT increases migration, and differentiation of bone cells to the site of the irradiation, culminating in the increase of extracellular matrix secretion and mineralization (11,14,24). These biological modifications may produce an increase in BMD may constitute an extra stimulus to bone formation (15).

The morphometric analysis revealed that diabetic animals showed a significantly decrease in total area and cortical area. It is well known that type 1 diabetes appears to revert active osteoblasts into inactive bone-lining cells and decrease proliferation of preosteoblastic cells (25). As a result, DM is marked by a significant decrease in bone formation, which is resp**onsible by the lower bone area and bone length (25). Furthermore, in the present study, the resistance training (both treated and non-treated) was able of increasing cortical area** in diabetic animals. These results corroborate those of Gomes and cols. (10) who observed that a swimming training increased the tibial length, total area and BMC in diabetic rats. Interestingly, the association of physical exercise and LLLT did not produce an additional effect on morphological aspects of bone.

RUNX2 is essential to osteoblast differentiation and osteogenesis and regulates the expression of many extracellular matrix protein genes during bone cell differentiation (26). In the presence of DM, an impaired osteoblast function is observed, with a decrease in RUNX2 expression (5). The results of the present study demonstrated that the resistance training had an osteogenic potential, stimulating the expression of this immunomarker. However, LLLT was not able of improving RUNX2 expression in the trained animals. It is unclear at this stage why these results have occurred however it may be hypothesized that the focal intensity and energy output of the laser light during the 6 week-exercise protocol were not sufficient to stimulate the expression of RUNX2.

In the same way, RANK-L is a member of the tumor necrosis factor (TNF) cytokine family, which is a ligand for receptor activator of the nuclear factor κ-β (RANK) and osteoprotegerin (OPG). It works as a key factor for osteoclast differentiation and activation (19,22). Our imunohistochemical analysis demonstrated similar findings of RANK-L immunolabeling of all experimental groups. These findings suggest that the DM did not have any influence in the monocyte-macrophage lineage, including macrophages and osteoclasts, having a normal function in the process of bone turnover.

Three-bending test showed that DM induced a decrease of bone biomechanical properties. Many studies have demonstrated that diabetic bones present lower mainly because of dysfunction in bone cell formation, leading to a lower bone mechanical strength (27). Furthermore, higher values of fracture force were observed in both trained groups and higher values of stiffness were observed in TLG. There is a lack in the literature investigating the effects of physical exercise programs in bone biomechanical properties. It is proposed that the resistance training, with high loads increases bone remodeling and bone biomechanical properties in pathological conditions (28). These statements corroborate with our findings, which suggest that exercise and the association of both treatments were able of producing a tendency of improving bone strength.

DM significantly reduced densitometric values and the resistance exercise protocol was effective to increase BMD and BMC. Zhang and cols*.* (27) have demonstrated a decreased femoral BMD in diabetic animals compared to normal animals. Additionally, Mathey and cols. (29) observed that a treadmill exercises protocol, increased BMD in obese diabetic rats. Interestingly, BMD of the irradiated trained animals was increased compared to TG. This approach is new in the literature and the results are difficult to discuss. It can be suggested that osteoblasts cells in TLG were more active and they were able of synthesizing a higher amount of bone mass (independent of the number of cells).

The osteogenic potential of LLLT has been demonstrated by many studies (13,15). However, the existence of window specificity at certain wavelengths and energy dosages also is well known (12,13,15). It means that a proper energy needs to be offered to the tissue in order to determine physiological modifications and consequently, stimulatory effects to the tissue (18). These indicate that, although some of the positive effects of the association of physical exercise and LLLT on bone, the application of laser, used with different parameters on the experimental conditions of this study should be investigated. Moreover, as this study was limited to relatively short-term evaluation of the effects of physical exercise and only one laser fluency, information on the long-term performance of these therapies and higher amount of laser energy remain to be provided.

In conclusion, it can be suggested that the resistance exercise program stimulated bone metabolism, culminating in increased cortical tibial area, bone mineral content, bone mineral density and biomechanical properties. Furthermore, the association of physical exercises and LLLT favored raise of the bone mineral content and stiffness. Consequently, these data highlight the potential of physical exercise in the management of bone loss due to DM and the possible extra osteogenic stimulus offered by lasertherapy.
